# Association of anthropometric indices with rs9939609 *FTO* gene polymorphism among overweight/obese women with breast cancer: a case-control study

**DOI:** 10.3389/fnut.2025.1548340

**Published:** 2025-06-24

**Authors:** Huma Naqeeb, Bismillah Sehar, Sami Siraj, Ali Madi Almajwal, Bibi Hajira, MoezAlIslam Faris, Sharifa AlBlooshi, Ali Saad R. Alsubaie, Mahpara Safdar, Iftikhar Alam, Falak Zeb

**Affiliations:** 1 Department of Human Nutrition and Dietetics, Women University Mardan, Mardan, Pakistan; 2 Faculty of Health, Education and Life Sciences, Birmingham City University, Birmingham, United Kingdom; 3 Institute of Pharmaceutical Sciences, Khyber Medical University, Peshawar, Pakistan; 4 Department of Community Health Sciences, College of Applied Medical Sciences, King Saud University, Riyadh, Saudi Arabia; 5 Department of Clinical Nutrition, Institute of Basic Medical Sciences, Khyber Medical University, Peshawar, Pakistan; 6 Department of Clinical Nutrition and Dietetics, Faculty of Allied Medical Sciences, Applied Science Private University, Amman, Jordan; 7 College of Natural and Health Sciences, Zayed University, Dubai, United Arab Emirates; 8 Department of Public Health, College of Public Health, Imam Abdulrahman Bin Faisal University, Dammam, Saudi Arabia; 9 Department of Environmental Design, Health and Nutritional Sciences, Allama Iqbal Open University, Islamabad, Pakistan; 10 Department of Human Nutrition and Dietetics, Bacha Khan University Charsadda, Peshawar, Pakistan; 11 Research Institute of Medical and Health Sciences, University of Sharjah, Sharjah, United Arab Emirates

**Keywords:** nutrigenomics, gene, anthropometric indices, breast cancer, fat mass and obesity

## Abstract

**Background/objectives:**

Fat mass and obesity associated (FTO) gene and anthropometric measurements might be associated with breast cancer (BC) risk. This study aimed to assess the interactions between single nucleotide polymorphism (SNP) rs9939609 of the FTO gene, anthropometric indices, and BC risk among pre- and post-menopause women with overweight/obesity in Pakistan.

**Methods:**

This retrospective case–control study conducted on a convenience sample of 200 women divided into two groups: a case group comprised of 100 women diagnosed with BC, and control group comprised of 100 (age and menopausal status matched healthy women). Physical activity was assessed using validated questionnaire. Data on body mass index (BMI, kg/m2), waist-to-hip ratio (WHR, cm), sociodemographic, and blood samples were collected from both groups. The rs9939609 FTO gene polymorphism was genotyped using the tetra-primer amplification refractory mutation system polymerase chain reaction and Sanger sequencing. Multiple regressions were presented as adjusted odds ratios (OR) and their respective confidence intervals (95% CI).

**Results:**

The FTO rs9939609 T > A polymorphism showed a significantly higher frequency of the homozygous AA genotype in BC patients compared to healthy controls (22% vs. 13%, *p* < 0.05). The odds ratio for BC was 2.4 (CI = 1.09–5.3, *p* < 0.05), indicating that women with the AA genotype were more susceptible to BC compared to those with the wild-type TT genotype. Additionally, BC patients exhibited significantly higher BMI (27 ± 4.0 vs. 25 ± 3.4, *p* < 0.05) and WHR (0.88 ± 0.06 vs. 0.85 ± 0.08, *p* < 0.05) compared to healthy controls. These findings suggest a significant association between the FTO rs9939609 AA genotype, obesity, and BC risk.

**Conclusion:**

FTO gene polymorphism may be implicated in the etiopathogenesis of BC, both in FTO pre- and post-menopause women diagnosed with overweight/obesity. Future cohorts are required to confirm the association between the FTO gene and BC in obese women and to identify the underlying biological mechanisms.

## 1 Introduction

Breast cancer (BC) is a group of diseases in which errors in cell division lead to a lump or tumor in the breast (1). It is the most frequently diagnosed cancer, with an incidence rate of 10.4% of all cancers, and responsible for about 1 million out of 10 million new neoplasms diagnosed every year around the world (2, 3). Pakistan has the highest rate of BC in Asia, with 1 in 9 women estimated to have a chance of developing BC at any stage of life (4). In Pakistan, the reported cases of BC were 25,928, which accounted for 14.5% of all types of cancer in 2020 (5).

Obesity has been reported to increase the risk of BC (6), and there is evidence that weight loss, as well as a decrease in fat intake, may reduce the risk of BC (7). The fat mass and obesity-associated (*FTO*) gene has lately emerged as one of the most widely studied genes linked to body mass. The *FTO* gene, also known as a 2-oxoglutarate dependent non-heme deoxygenate family member, is in the nucleus and spans more than 400Kb on chromosome 16, and it has nine exons (8). Given the strong association of obesity with BC, several studies examined the relationship between *FTO* gene polymorphism and the incidence of BC (9). Thus, single nucleotide polymorphism (SNP) of *FTO* has been associated with an increased risk of obesity in genome-wide association studies (GWAS) (10). Homozygous loss-of-function of the *FTO* gene was reported to cause severe growth retardation and multiple malformations (11). In contrast, a duplication of the *FTO* gene was found to be associated with morbid obesity (12).

The prevalence of the *FTO* rs9939609 polymorphism varies significantly across different populations. The A-allele, associated with increased susceptibility to obesity, has been observed in higher frequencies in European populations, where approximately 40–50% of individuals carry at least one copy of the A-allele (13). In contrast, the prevalence is lower in East Asian populations, ranging from 12–20% (14, 15). Another study reported that approximately 30–40% of South Asian populations, including populations in India and Pakistan, carry the A-allele (16). The frequency of the AA genotype is similarly variable, with higher prevalence rates reported in Caucasian populations compared to African and East Asian populations (17). Further studies have documented the impact of the *FTO* genotype on body weight and obesity traits. Mehrdad et al., observed that individuals with the AA genotype had significantly higher body weight (~3.6 kg and ~10.1 kg more, respectively) compared to those with the AT and TT genotypes (18). Moreover, carriers of the A-allele exhibited higher BMI and fat mass (FM) than non-carriers (18). Andreasen et al., in a large Danish cohort, found that homozygous A-allele carriers had a 1.1 kg/m^2^ higher BMI and 2.3 cm greater waist circumference (WC) compared to their non-carrier counterparts (19). Similarly, a meta-analysis conducted by Kilpeläinen et al. (20) confirmed that individuals with the AA genotype had a 1.67-fold increased risk of obesity across multiple European populations.

In South Asian populations, the association between *FTO* polymorphisms and obesity has been studied, with results indicating a significant correlation between the A-allele and increased adiposity measures (21). A study conducted in India by Vimaleswaran et al. (22) reported that the presence of the A-allele associated with a higher risk of central obesity. In addition to the widely studied rs9939609 variant of the *FTO* gene, other SNPs such as rs8050136 and rs1558902 have also been shown to be significantly associated with obesity-related traits. These SNPs are located within the same region of the *FTO* gene and have been implicated in influencing BMI, WC, and fat mass (23). Studies suggest that the rs8050136 SNP shares a strong linkage disequilibrium with rs9939609 and exerts a similar influence on obesity risk, while rs1558902 has been linked to higher fat accumulation and increased obesity risk in various populations. There is growing evidence that dietary and lifestyle modifications can significantly influence the expression of genes, including the *FTO* gene, which plays a critical role in energy homeostasis and obesity (24). Modifications such as increased physical activity and specific dietary interventions have been shown to reduce *FTO* gene expression, potentially lowering obesity risk (24). These genetic factors, combined with nutritional influences, can help explain the variability in the prevalence and severity of breast cancer, as well as other chronic diseases. However, the possible association between nutrition and genetics in the context of BC is not clearly elucidated, particularly in developing societies like Pakistan, where both nutrition and genetics are more diverse than in the rest of the world.

Obesity is a well-established risk factor for breast cancer (BC), with increasing evidence linking genetic factors, particularly the *FTO* gene, to obesity and BC susceptibility (13, 25). Notably, the rs9939609 SNP has been widely studied in relation to body mass index (BMI) and waist-to-hip ratio (WHR), both of which are important anthropometric measures linked to BC risk (26). The rs9939609 variant has been reported to be present in varying frequencies across different ethnic groups, with studies indicating its prevalence in populations of South Asian descent (27). This study specifically focuses on the *FTO* rs9939609 SNP, as its association with obesity and its role in BC risk, especially in populations with high rates of obesity, remains underexplored. Given the growing prevalence of obesity in Pakistan and the lack of comprehensive studies examining genetic and anthropometric interactions in relation to BC risk, this SNP was chosen to investigate its potential contribution to BC susceptibility in Pakistani women with overweight/obesity.

## 2 Materials and methods

### 2.1 Study design and population

This was a retrospective case–control study conducted at two tertiary care facilities in the Khyber Pakhtunkhwa (KP) region of Pakistan including Oncology Units’ Outpatients Department (OPD) of the Institute of Radiation and Nuclear Medicine (IRNUM), and Khyber Teaching Hospital (KTH). STROBE guidelines have been followed for this study (28). Ethical approval was obtained from the Faculty of Nutrition Sciences, Ethics Committee and Human Studies Review Board (FNS-ECHSRB-2020-02) at The University of Agriculture, Peshawar, Pakistan.

### 2.2 Sample size

The sample size was determined using Epi Info, following established guidelines (29). The calculation assumed a 70% exposure probability among controls and a 0.2 correlation coefficient between matched cases and controls. With a true odds ratio of 3 for the disease in exposed versus unexposed individuals, the required sample size for this case–control study was 200 participants (100 cases and 100 controls). This calculation was based on achieving 80% power and 5% types I error rate for testing the null hypothesis. The study employed a convenience sampling method, which may limit the external validity as the sample may not fully represent the broader population, potentially affecting the generalizability of the results to different settings or populations. To address this, the sample was selected from a specific region, aiming to capture a cohort reflective of the local population.

### 2.3 Groups allocation and criteria

Upon assessment, the oncologist confirmed the histology and the presence of initial BC in newly diagnosed women with overweight and/or obesity. The same menopause status and age match (±5.0 years) were the prerequisites of the hospital and visitor-based control (healthy) for assuring to have similar racial/genetic, economic, and cultural backgrounds of both groups. Written consent from the subjects was taken prior to the commencement of the study. The eligible women divided into two groups: a case group comprised of 100 women diagnosed with BC, and healthy control group comprised of 100, age and menopausal status matched healthy women. The inclusion criteria for both groups were women aged 20–65 years, while exclusion criteria included those with genetic disorders, pregnancy, breastfeeding, and other comorbidities.

### 2.4 Sociodemographic data

Data on socioeconomic status, including education, occupation status, and lifestyle factors (such as physical activity) were collected using a self-administered questionnaire.

### 2.5 Reproductive characteristics

Reproductive history regarding the age at first menarche, age at first pregnancy, parity, usage of oral contraceptives, and hormone replacement therapy were questioned. Participants were also asked about any associated co-morbidities or family history of BC and their responses were noted in written form. The matching technique involved pairing participants in the case and control groups by age (within a 5-year range), and menopausal status (pre- or post-menopausal), which was operationalized based on self-reported age and menstrual history to control for hormonal differences that could influence the outcomes.

### 2.6 Anthropometric measurements and physical activity

Using standardized instruments, women’s anthropometric measurements such as weight, height, and waist and hip circumferences were undertaken (30). Weight and height measurements were used to compute BMI (kg/m^2^), while women’s waist and hip measurements were used to determine waist-to-hip ratio (WHR, cm) (31). For physical activity, the International Physical Activity Questionnaire (IPAQ). These tools have been previously validated in similar studies, ensuring their reliability (32).

### 2.7 Blood sample collection

Blood samples were collected in phlebotomy under the standard guidelines through trained nurses after approval from hospital administration. Each subject was drawn approximately 3 mL of blood from the antecubital vein. Tubes containing 3 mL EDTA were used for DNA isolation and subsequent gene polymorphism determination. Samples were collected and kept at-20°C in the refrigerator in the Pharmacogenomics laboratory-Khyber Medical University-Peshawar.

### 2.8 Genotyping/allele-specific PCR and sequencing

DNA was extracted using the ThermoScientific #K0781 kit and stored at-20°C. To amplify the DNA fragment containing the *FTO* gene polymorphism (rs9939609), a polymerase chain reaction (PCR) was set up with the following components: 1 μL of 2.5 mM dNTPs, 2 μL of NH4SO4 buffer, 25 mM MgCl2, 0.8 μL of 10 μM forward primer (5′-TGGAGAATGATGAAGTA-3′), 0.7 μL of 10 μM reverse primer (5′-GCAATTTAAGTAATGCCTAT-3′), 0.7 μL of Taq polymerase, 1.5 μL of DNA template, and 12.5 μL of deionized water, all mixed in a 20 μL volume. The PCR conditions included an initial denaturation at 95°C for 5 min, followed by 35 cycles of 95°C for 30 s (denaturation), 68°C for 45 s (annealing), and 72°C for 45 s (extension), with a final extension at 72°C for 10 min.

For gel electrophoresis, a 2% agarose gel was prepared, and 20 μL of each amplified DNA sample was mixed with four μL of 6x loading dye. The gel was run at 90 V for 30 min. Bands were visualized under UV light to determine genotypes. For sequencing, the purified DNA samples were sent to TSINGKE Biotechnology Co., Ltd. in China for Sanger sequencing. The genotypes were confirmed by comparing sequencing results with those from allele-specific PCR using online tools like BLAST and Clustal W.

### 2.9 Statistical analysis

Data were analyzed using SPSS software version 20 (IBM). Comparisons between the two groups were made regarding demographics, reproductive history, anthropometric measurements, and the presence of the *FTO* gene polymorphism. Initially, t-tests were used for quantitative variables and chi-square tests for qualitative variables. We performed logistic regression analysis to explore the association between FTO polymorphisms and breast cancer (BC), adjusting for key covariates that could influence the results. These included age, menopausal status, hormonal replacement therapy (HRT) use, oral contraceptive use, family history of breast cancer, physical activity level. These factors were selected based on their established impact on BC risk and obesity. The logistic regression models were first performed unadjusted, followed by adjustments for these covariates to account for potential confounding effects. This approach ensured that the association between FTO polymorphisms and BC was independent of other known risk factors. To assess the relationship between BC and the risk allele of the rs9939609 polymorphism, logistic regression analysis was conducted. Results were reported as odds ratios (OR) with 95% confidence intervals (CI), and significance was set at *p* < 0.05. Genotype percentages and frequencies were calculated for Hardy–Weinberg equilibrium using Microsoft Excel 2010. Deviations from Hardy–Weinberg equilibrium were tested using the chi-square test and the “OEGE Online Hardy–Weinberg Equilibrium Calculator”.

## 3 Results

### 3.1 Descriptive characteristics of the BC patients (case vs. control)

The Table 1 presents general characteristics of individuals encompassing 816 participants. There was no significant variation among the ages of the studied participants and majority of participants were married (90%). While there was a slight trend suggesting higher odds of being a BC among single individuals, this association was not statistically significant after adjusting for confounders. There was no significant association between family type and BC status after adjustment. Similarly, family size categories did not demonstrate a significant association with BC and healthy status after adjustment. The education level emerged as a significant predictor, with illiterate individuals showing 2.8 times higher odds of being BC compared to literate individuals after adjusting for potential confounders. These findings underscore the importance of considering socio-demographic factors in understanding and addressing the risk factors associated with the outcome under investigation. Breastfeeding practices also revealed a significant association, with those who breastfed having lower odds of being BC risk (OR = 0.6, 95% CI: 0.2–1.7) after adjustment. Additionally, the menopausal status displayed a noteworthy correlation, with postmenopausal women over 45 years old exhibiting increased odds of being BC (OR = 2.9, 95% CI: 1.5–3.9) after adjusting for confounders. Hormone replacement therapy (HRT) and oral contraceptives (OC) use demonstrated associations as well, with OC users showing higher odds of being BC (OR = 2.6, 95% CI: 1.4–3.5) after adjustment. In contrast, no such significant association was found for HRT after adjustment. These findings emphasize the complex relationship of reproductive factors in the context of BC risk, necessitating comprehensive understanding and targeted interventions in cancer prevention and management strategies.

**Table 1:** Descriptive characteristics of the BC patients (*n* = 200).

Characteristics		Mean ± SD/*N*%			*P*-value	Unadjusted	Adjusted**
		Total	BC	Healthy			
Age		200	45 ± 6.3	45 ± 3.3	> 0.05	–	–
Marital status	Single	23	7 (7%)	16 (16%)	> 0.05	Reference	Reference
	Married	177	93 (93%)	85 (85%)		0.7 (0.4–1.2)	0.9 (0.6–1.5)
Menopause status	Postmenopausal	101	51 (51%)	50 (50%)	<0.05	Reference	Reference
	Premenopausal	99	50 (50%)	49 (49%)		1.4 (1–1.7)*	2.9 (1.5–3.9)*
Hormones replacement therapy (HRT)	Never	176	85 (85%)	91 (91%)	>0.05	Reference	Reference
	Yes	14	15 (8%)	6 (9%)		1.5 (0.9–2.5)	1.4 (0.5–2.75)
Oral contraceptive (OC) duration	Never	178	86 (86%)	92 (90%)	<0.05	Reference	Reference
	yes	22	14 (10%)	7 (7%)		1.9 (1.2–3.2)*	2.6 (1.4–3.5)*
Family history of BC	No	172	84 (84%)	88 (88%)	0 > 0.05	Reference	Reference
	Yes	28	16 (16%)	12 (12%)		1.7 (1.1–2.6)*	1.8 (0.7–2.6)
Physical activity	No	160	86 (86%)	74 (74%)	>0.05	Reference	Reference
	Yes	40	14 (7%)	16 (16%)		0.04 (0.2–0.6)*	0.7 (0.2–2)

SD = standard deviation; *N*% = number of women (percentage), * At α = 0.05, to compare the two groups, student *t*-tests and chi-square tests were used, **adjusted with marital status as single, HRT and OC usage, family history of BC, and, breast-feeding practice.

### 3.2 Anthropometric status and risk of breast cancer

Table 2 presents the anthropometric data of individuals, including BMI classification, WC, and WHR, and their relation to BC risk with both unadjusted and Adjusted ORs. After adjustment, underweight individuals showed a non-significant decrease in BC risk compared to those with normal BMI. Those with a higher WC (>80 cm) also showed a non-significant increase of BC risk. However, individuals with a higher WHR (>0.85) exhibited a significant increase in BC risk compared to those with normal WHR (<0.85) after adjustment (Adj. OR = 2.2, 95% CI: 1.2–3.9), suggesting that abdominal obesity may be associated with increased risk of BC.

**Table 2:** Anthropometric status of both case and control groups (*n* = 200).

BMI classification	Total	BC	Healthy	*p* value	OR (95%CI)	
					Unadjusted	Adjusted**
Normal (18.5–24.9)	57	24 (24%)	33 (33%)	>0.05	Reference	Reference
Underweight (<18.5)	26	15 (15%)	11 (11%)		0.4 (0.3–1.)	0.6 (0.5–1.1)
Overweight (25–29.9)	72	35 (35%)	37 (37%)	>0.05	1.2 (0.8–1.4)	1.1 (0.9–1.3)
Obese (>30)	45	(26%)	19 (19%)	<0.001	1.0 (1–1.5)*	1.01 (1–1.04)*
WC classification						
Normal (≤80 cm)	89	41 (41%)	48 (48%)	>0.05	Reference	Reference
High WC (>80 cm)	111	59 (59%)	52 (52%)		1.5 (0.9–1.8)	1.4 (0.9–2.2)
WHR Classification						
Normal WHR	142	68 (68%)	74 (74%)	<0.05	Reference	Reference
High WHR (>0.85)	58	32 (32%)	26 (26%)		1.3 (1–1.9)*	2.2 (1.2–3.9)*

*At α = 0.05, Student t tests or chi-square tests were used to compare group differences. **Age at fist menarche, age at first pregnancy, menopause status, WHR = waist to hip ratio.

### 3.3 FTO gene polymorphism in women with BC and healthy control

**Figure 1:**
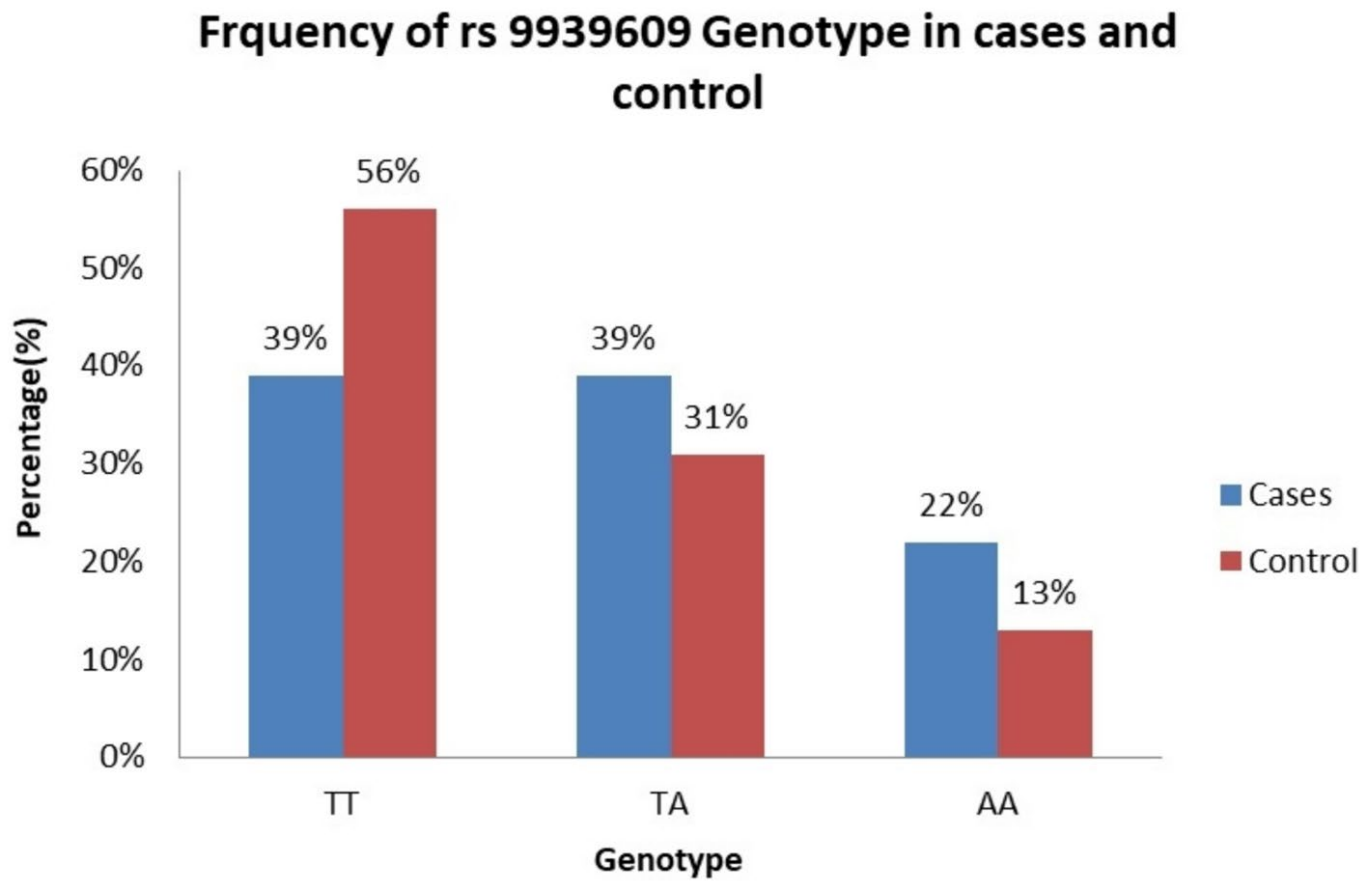
Frequency of rs9939609 genotype. TT genotype is more common in controls. AA genotype is more common in BC group, aligning with the statistically significant adjusted OR (2.4, *p* < 0.05). TA genotype is equally distributed in BC group but lower in healthy control.

Table 3 presents the frequency distribution of the genotypes and alleles at the *FTO* gene rs9939609 polymorphism in women with BC and healthy controls. Among the BC group, the TT genotype was observed in 39% of women, the TA genotype in 39%, and the AA genotype in 22% (Figure 1). In the healthy control group, the TT genotype was more prevalent (56%), followed by the TA genotype (31%) and the AA genotype (13%) (Figure 1). The analysis revealed that women carrying the TA genotype had an increased but not statistically significant risk of BC, with an adjusted OR of 1.8 (95% CI: 0.96–5.3) compared to those with the TT genotype. However, the AA genotype was associated with a significant increased risk of BC, with an adjusted OR of 2.4 (95% CI: 1.09–5.3), indicating that women with the AA genotype were more likely to develop BC than those with the TT genotype.

**Table 3:** The frequency of genotypes and alleles at the *FTO* gene in case and control.

rs 9939609	BC (%) (*n* = 100)	Healthy (%) (*n* = 100)	Adjusted OR (95%CI)
Genotyping			
TT	39 (39%)	56 (56%)	Ref
TA	39 (39%)	31 (31%)	1.8 (0.96–5.3)
AA	22 (22%)	13 (13%)	2.4 (1.09–5.3)*

Adjusted for Age, Menopause status, HRT and OC usage & BMI, *P value <0.05.

### 3.4 Hardy–Weinberg equilibrium (HWE) analysis

Table 4 presents the genotyping distribution of the *FTO* rs9939609 polymorphism in BC (BC) patients and healthy controls, along with the Hardy–Weinberg equilibrium (HWE) analysis for both groups. In the BC group, the observed frequencies of the TT, TA, and AA genotypes were 39, 39, and 22%, respectively, while the expected frequencies were 34% for TT, 48% for TA, and 17% for AA. The chi-square (χ^2^) value for HWE in the BC group was 3.8, with a *p*-value of 0.04, indicating a significant deviation from Hardy–Weinberg equilibrium. In the healthy control group, the observed frequencies for the TT, TA, and AA genotypes were 56, 31, and 13%, respectively, while the expected frequencies were 51% for TT, 40% for TA, and 8% for AA. The chi-square (χ^2^) value for HWE in the control group was 5.7, with a *p*-value of 0.01, also indicating a significant deviation from Hardy–Weinberg equilibrium. These results suggest that the genotype distribution of the *FTO* rs9939609 polymorphism has significant deviations from HWE in both groups suggest that the population might not be in equilibrium for the rs9939609 polymorphism. This could be due to several factors, such as non-random mating, genetic drift, selection pressure, or population stratification, and small sample size.

**Table 4:** Genotyping distribution of *FTO* rs9939609 for HWE between case and healthy control (*n* = 200).

Genotype	BC (*n* = 100)		HWE		Healthy (*n* = 100)		HWE	
	Observed value	Expected value	X^2^	*P*-value	Observed value	Expected value	X2	*P*-value
TT	39	34	3.8	<0.05	56	51	5.7	0.01
TA	39	48			31	40		
AA	22	17			13	8		

### 3.5 Association of FTO genotypes with obesity

Table 5 shows the association of *FTO* rs9939609 genotypes with obesity, measured by Body Mass Index (BMI) and Waist-Hip Ratio (WHR), in women with BC and healthy controls. For the TT genotype, the mean BMI was similar between BC and healthy control group (24 ± 2.9 vs. 24 ± 3.4), with no statistically significant difference. The TA genotype showed significant difference in BMI between cases and controls (26 ± 1.6 vs. 25 ± 3.3). Also, women with the AA genotype had a significantly higher BMI in BC compared to control (27 ± 4.0 vs. 25 ± 3.4, *p* < 0.05). Regarding WHR, no significant differences were observed for the TT and TA genotypes between BC and healthy counterpart. The TT genotype showed similar WHR values (0.85 ± 0.00 vs. 0.84 ± 0.08), and the TA genotype also showed comparable WHR values (0.86 ± 0.08 vs. 0.85 ± 0.09). In contrast, the AA genotype was associated with a significantly higher WHR in BC compared to healthy control (0.88 ± 0.06 vs. 0.85 ± 0.08, *p* < 0.05). These findings suggest that the AA genotype of the *FTO* rs9939609 polymorphism may be linked to increased obesity risk, as indicated by higher BMI and WHR, in women with BC.

**Table 5:** Association of *FTO* genotypes with obesity in case and healthy control.

VDR SNPs	Genotype	Mean BMI			Adjusted *p*-value
		Total (*N*)	BC	Healthy	
*FTO* rs9939609	TT	23	24 ± 2.9	24 ± 3.4	>0.05
	TA	142	26 ± 1.6	25 ± 3.3	<0.00
	AA	35	27 ± 4.0	25 ± 3.4	<0.05
Mean WHR (>0.85)					
*FTO* rs9939609	TT	21	0.85 ± 0.00	0.84 ± 0.08	>0.05
	TA	139	0.86 ± 0.08	0.85 ± 0.09	>0.05
	AA	40	0.88 ± 0.06	0.85 ± 0.08	<0.05

The adjusted *p*-values considering the following covariates: age, menopausal status, hormonal replacement therapy, oral contraceptive use, history of breast cancer, and physical activity. Adjustments were made using logistic regression analysis.

The inclusion of these covariates was based on their established association with both obesity and breast cancer, ensuring that the results for the *FTO* polymorphism were not confounded by other factors. All models were adjusted for these variables to provide a clearer understanding of the independent effect of the *FTO* rs9939606 polymorphism on BC risk.

## 4 Discussion

The present case–control study identified an association between BC and the risk allele of the *FTO* rs9939609 polymorphism among Pakistani participants, the first of its kind study in Pakistan. The *FTO* gene is a novel gene, and SNPs in *FTO* have been linked to an increased risk of obesity in genome-wide association studies (GWAS). We explored the correlation between *FTO* polymorphism and BC risk associated with high BMI, and WHR in both cases and healthy control groups. Among women with a BMI > 24.9 and a WHR > 0.85, we observed a significant difference in the distribution of *FTO* polymorphism between cases and healthy controls. This suggests that abnormal anthropometric indices may increase the risk of BC, which in turn may influence the polymorphism of the *FTO* gene.

The association between *FTO* gene polymorphism and BC has been studied across different ethnic groups with varying results. Findings of the current study suggest that hormone replacement treatment (HRT) increases the risk of BC. This is supported by Li et al., 34, who suggested that the combination of estrogen and ER positivity in developing mammary gland promotes tumor growth and angiogenesis, which is one of the mechanisms by which HRT & OC (oral contraceptive) use affects BC in younger women (33). Recent studies continue to emphasize the significant impact of family history on BC risk. Women with a first-degree relative diagnosed with BC are at approximately double the risk of developing the disease themselves (35). Although hereditary BC cases account for only 5–10% of all BC cases, updated estimates suggest that by the age of 70, 55–72% of *FTO* mutation carriers will develop BC. These findings reinforce the importance of genetic counseling and testing for women with a family history of BC (36). The results of this study found a highly significant association between physical activity and reduced BC (BC) risk. These findings are consistent with previous research, which also indicates that regular physical exercise (3–5 days a week) can reduce the risk of BC by 20–40% (37). Recent studies further support that physical activity lowers estrogen levels and other growth factors, contributing to a decreased risk of BC. This protective effect underscores the importance of incorporating regular physical activity into lifestyle recommendations for BC prevention (38).

Regarding the rs9939609 SNP of the *FTO* gene, our study results align with recent findings that suggest a significant correlation between this polymorphism and an increased risk of BC, particularly in certain populations. Recent research by Li et al. (34) has demonstrated that the rs9939609 polymorphism is associated with higher BMI and increased BC risk, particularly in postmenopausal women (39). The biological mechanism underlying this association may involve the role of the *FTO* gene in adipogenesis and energy balance regulation. The *FTO* gene influences the demethylation of N6-methyladenosine (m6A) in RNA, which affects the expression of genes involved in lipid metabolism and fat storage. This can lead to increased adiposity, higher estrogen levels, and insulin resistance, all of which are risk factors for BC. The study further elaborated that the AA genotype is associated with higher levels of circulating estrogen, which may contribute to tumor growth in estrogen receptor-positive BC (40).

Recent studies have highlighted a significant association between *FTO* gene polymorphisms, particularly the rs9939609 variant, and breast cancer risk, with findings showing variability across different populations and metabolic contexts. For instance, research conducted on European and Asian cohorts demonstrates that the AA genotype of the *FTO* rs9939609 polymorphism is linked to an elevated risk of breast cancer, potentially due to its influence on BMI, hormonal changes, and energy metabolism (41). Furthermore, a meta-analysis of multiple studies across various ethnic groups emphasizes that *FTO’s* role in breast cancer risk is often mediated by factors like obesity, estrogen receptor status, and metabolic health (42). Studies have consistently shown that the A allele of the *FTO* rs9939609 variant is strongly linked to higher BMI and increased obesity risk. For instance, research conducted in Chinese and South Asian populations has demonstrated that carriers of the A allele experience higher risks of obesity and type 2 diabetes (43). Additionally, meta-analyses across various cohorts from Europe, Latin America, and Asia have confirmed the role of the A allele in contributing to elevated BMI and obesity susceptibility (44). This aligns with findings that *FTO* gene variations influence energy regulation, appetite, and metabolism, underscoring their impact on obesity across different ethnic and demographic groups. These studies collectively validate the strong connection between *FTO* polymorphisms and obesity, emphasizing the need for targeted genetic research and intervention strategies in public health initiatives globally (45).

In contrast, a study on the Iranian population, involving 1,134 cases and 1,453 controls, found no significant association between rs9939609 and BC risk (46). However, supporting our findings, a study also reported that the rs9939609 *FTO* gene polymorphism significantly increases the risk of BC in overweight and obese women (45). This study highlighted that increased adiposity associated with the AA genotype could enhance estrogen production from adipose tissue, thereby promoting estrogen-dependent tumor growth in breast tissue (47). Several biological mechanisms link obesity and BC risk, such as obesity increases insulin resistance, hyperinsulinemia, growth hormone secretion, the production of carcinogens (mitogenesis, mutagenesis, angiogenesis, reduced apoptosis, metastasis, and immunosuppression), oxidative stress, and the inflammatory processes (48). For the justification of the mechanistic role of *FTO* in metabolism, Liu et al. assessed the effect of *FTO* on the energy metabolism of BC cells. They explained the mechanism as an *FTO* inhibitor restrained pyruvate kinase and hexokinase activity and suppressed BC cell glycolysis, partly through lowering the levels of PI3K, p-PI3K, Akt, and p-Akt, which were members of PI3K/AKT signaling pathway and progress tumor growth (49). It was also revealed by Gholamalizadeh et al. that *FTO* functioned to activate the PI3K/Akt signaling pathway and promote BC cell proliferation in BC patients (50). Furthermore, P53 is a tumor suppressor gene that encodes a nuclear phosphoprotein that is known to control normal cell growth. P53 mutations produce a nonfunctional protein that accumulates in tumor cell nuclei and appears to have a role in the development and/or progression of a variety of cancers, including human BC (51).

Obesity has been established as a significant risk factor for BC. Evidence suggests that weight loss and reductions in fat consumption can lead to a decreased risk of developing BC (11). The *FTO* gene, which is associated with obesity, has been linked to various health conditions through its role in regulating body weight and metabolism. Homozygous loss-of-function mutations in *FTO* have been associated with severe growth retardation and multiple malformations, highlighting the gene’s critical role in normal development (52). Conversely, duplications of the *FTO* gene have been found to contribute to morbid obesity, further emphasizing its role in weight regulation and associated health risks (11). Furthermore, population stratification may have influenced genotype distributions, especially in a genetically diverse setting like Pakistan, potentially contributing to the deviation from Hardy–Weinberg Equilibrium (HWE) observed in both case and control groups. Such deviation could reflect underlying population substructure or sampling bias, and thus the genetic associations observed should be interpreted cautiously. While this study supports a potential association between FTO rs9939609 polymorphism, obesity, and breast cancer (BC), the mechanistic pathways linking these variables remain speculative and require further validation in longitudinal or functional studies. Additionally, unmeasured confounders such as physical activity, and socioeconomic status—factors known to influence both BMI and BC risk (53, 54)—may have impacted the associations observed and should be considered in future research for more robust conclusions.

Limitations of the study including, first that other anthropometric measurements, such as body fat composition, were not assessed. Second, this study was limited to only one SNP of the *FTO* gene, and other SNPs of obesity gene may have different associations with BC. Finally, further studies on the risk of FTO gene polymorphism with BC will be required on a larger sample size.

## 5 Conclusion

This study might suggest that the *FTO* rs9939609 AA genotype is associated with increased obesity in both pre and post-menopause women with BC; it could be more clarified with a large sample size. This genetic association highlights the potential role of *FTO* in obesity-related cancer risk. Further research is needed to explore the biological mechanisms underlying this association and to develop targeted interventions for individuals with genetic predispositions to both obesity and cancer. Future research is required to confirm the link between the *FTO* gene and BC in obese females, as well as to expose the mechanisms that underlie it.

## Data Availability

The raw data supporting the conclusions of this article will be made available by the authors, without undue reservation.

## References

[1] Smith RA Andrews KS Brooks D Fedewa SA Manassaram-Baptiste D Saslow D . Cancer screening in the United States, 2018: a review of current American Cancer Society guidelines and current issues in cancer screening. CA Cancer J Clin. (2018) 68:297–316. doi: 10.3322/CAAC.21446, PMID: 29846940

[2] Torre LA Islami F Siegel RL Ward EM Jemal A. Global cancer in women: burden and trends. Cancer Epidemiol Biomarkers Prev. (2017) 26:444–57. doi: 10.1158/1055-9965.EPI-16-0858 28223433

[3] Bray F Ferlay J Soerjomataram I Siegel RL Torre LA Jemal A. Global cancer statistics 2018: GLOBOCAN estimates of incidence and mortality worldwide for 36 cancers in 185 countries. CA Cancer J Clin. (2018) 68:394–424. doi: 10.3322/CAAC.21492, PMID: 30207593

[4] Mustafa A KUR Khalil Bashirullah N Iftikhar B. Risk factors of breast cancer in female at IRNUM cancer hospital, Peshawar. J Med Sci (2017) 25:273–278. Availabe online at: https://jmedsci.com/index.php/Jmedsci/article/view/65

[5] Hussain I Majeed A Masood I Ashraf W Imran I Saeed H . A national survey to assess breast cancer awareness among the female university students of Pakistan. PLoS One. (2022) 17:e0262030. doi: 10.1371/JOURNAL.PONE.0262030, PMID: 35061770 PMC8782286

[6] World Cancer Research Fund (WCRF): Continuous update. Google scholar. Available online at https://scholar.google.com/scholar_lookup?title=Continuous Update Project: 2016&publication_year=2016& (Accessed December 13, 2024)

[7] Cheung M Gulati P O’Rahilly S Yeo G. S. H.. (2013). FTO expression is regulated by availability of essential amino acids. Int J Obes. Available online at: https://www.nature.com/articles/ijo201277 [Accessed December 13, 2024] 10.1038/ijo.2012.77 22614055

[8] Da Cunha PA De Carlos Back LK Sereia AFR Kubelka C Ribeiro MCM Fernandes BL . Interaction between obesity-related genes, FTO and MC4R, associated to an increase of breast cancer risk. Mol Biol Rep. (2013) 40:6657–64. doi: 10.1007/S11033-013-2780-3, PMID: 24091943

[9] Doaei S Mosavi Jarrahi SA Sanjari Moghadam A Akbari ME Javadi Kooshesh S Badeli M . The effect of rs9930506 FTO gene polymorphism on obesity risk: a meta-analysis. Biomol Concepts. (2020) 10:237–42. doi: 10.1515/BMC-2019-0025/HTML 31855561

[10] Chermon D Birk R. FTO common obesity SNPs interact with actionable environmental factors: physical activity, sugar-sweetened beverages and wine consumption. Nutrients. (2022) 14:4202. doi: 10.3390/nu14194202 36235854 PMC9572787

[11] Boissel S Reish O Proulx K Kawagoe-Takaki H Sedgwick B Yeo GSH . Loss-of-function mutation in the dioxygenase-encoding FTO gene causes severe growth retardation and multiple malformations. Am J Hum Genet. (2009) 85:106–11. doi: 10.1016/J.AJHG.2009.06.002, PMID: 19559399 PMC2706958

[12] Huang C Chen W Wang X. Studies on the fat mass and obesity-associated (FTO) gene and its impact on obesity-associated diseases. Genes Dis. (2023) 10:2351–65. doi: 10.1016/J.GENDIS.2022.04.014, PMID: 37554175 PMC10404889

[13] Frayling TM Timpson NJ Weedon MN Zeggini E Freathy RM Lindgren CM . A common variant in the FTO gene is associated with body mass index and predisposes to childhood and adult obesity. Science. (2007) 316:889–94. doi: 10.1126/SCIENCE.1141634, PMID: 17434869 PMC2646098

[14] Ng MCY Shriner D Chen BH Li J Chen WM Guo X . Meta-analysis of genome-wide association studies in African Americans provides insights into the genetic architecture of type 2 diabetes. PLoS Genet. (2014) 10:e1004517. doi: 10.1371/JOURNAL.PGEN.1004517, PMID: 25102180 PMC4125087

[15] Zhang J Feng JY Ni YL Wen YJ Niu Y Tamba CL . pLARmEB: integration of least angle regression with empirical Bayes for multilocus genome-wide association studies. Hered. (2017) 118:517–24. doi: 10.1038/hdy.2017.8, PMID: 28295030 PMC5436030

[16] Beruni SM Al. A review on single nucleotide polymorphism (SNPs) and their effect on cancer risk in south Asian population. (2021). Available online at: https://dspace.bracu.ac.bd:8443/xmlui/handle/10361/16175 (Accessed December 13, 2024)

[17] Peng XE Wu YL Lu QQ Hu ZJ Lin X. Two genetic variants in FABP1 and susceptibility to non-alcohol fatty liver disease in a Chinese population. Gene. (2012) 500:54–8. doi: 10.1016/J.GENE.2012.03.050, PMID: 22465531

[18] Mehrdad M Fardaei M Fararouei M Eftekhari MH. The association between FTO rs9939609 gene polymorphism and anthropometric indices in adults. J Physiol Anthropol. (2020) 39:1–7. doi: 10.1186/S40101-020-00224-Y/TABLES/4 32398148 PMC7218491

[19] Jess T Zimmermann E Kring SII Berentzen T Holst C Toubro S . Impact on weight dynamics and general growth of the common FTO rs9939609: a longitudinal Danish cohort study. Int J Obes. (2008) 32:1388–94. doi: 10.1038/ijo.2008.110, PMID: 18663371

[20] Kilpeläinen Tuomas O Zillikens M Carola Stančákova Alena Finucane Francis M Ried Janina S Langenberg Claudia ., (2011). Genetic variation near IRS1 associates with reduced adiposity and an impaired metabolic profile. Nat Genet. Available online at: https://www.nature.com/articles/ng.866 (Accessed December 14, 2024) 10.1038/ng.866 PMC3262230 21706003

[21] Ahmad S Fatima SS Rukh G Smith CE. Gene lifestyle interactions with relation to obesity, cardiometabolic, and cardiovascular traits among south Asians. Front Endocrinol (Lausanne). (2019) 10:221. doi: 10.3389/FENDO.2019.00221 31024458 PMC6465946

[22] Society KV-P of the N. (2020). A nutrigenetics approach to study the impact of genetic and lifestyle factors on cardiometabolic traits in various ethnic groups: findings from the GeNuIne. cambridge.org KS VimaleswaranProceedings Nutr Soc 2020•Cambridge.Org. Available online at: https://www.cambridge.org/core/journals/proceedings-of-the-nutrition-society/article/nutrigenetics-approach-to-study-the-impact-of-genetic-and-lifestyle-factors-on-cardiometabolic-traits-in-various-ethnic-groups-findings-from-the-genuine-collaboration/98F5A39BE96DAB0BA8612617E8DC3D4A (Accessed December 14, 2024) 10.1017/S0029665119001186 32000867

[23] Tan P Mitra S Genomics FA, (2019). Lifestyle interventions for weight control modified by genetic variation: a review of the evidence. Public Health Genom. Available online at: https://karger.com/phg/article-abstract/21/5-6/169/272925 [Accessed December 14, 2024] 10.1159/000499854 31117103

[24] Conway R Rockhold JD SantaCruz-Calvo S Zukowski E Pugh GH Hasturk H . Obesity and fatty acids promote mitochondrial translocation of STAT3 through ROS-dependent mechanisms. Front Aging. (2022) 3:924003. doi: 10.3389/FRAGI.2022.924003 35928250 PMC9344057

[25] Loos RJ Yeo GS. The genetics of obesity: from discovery to biology. Nat Rev Genet. (2014) 15:675–87. 10.1038/s41576-021-00414-z PMC8459824 34556834

[26] Shah P Ghaffar A Ahsan T. Association of FTO gene polymorphisms with obesity in south Asian populations. Obes Rev. (2020) 21:e13091

[27] Zhao JH Luan J Wu Y. Association of FTO rs9939609 polymorphism with obesity and body mass index in south Asian populations: a meta-analysis. Diabetes Metab. (2017) 43:35–42.

[28] von Elm E Altman DG Egger M Pocock SJ Gøtzsche PC Vandenbroucke JP. The strengthening the reporting of observational studies in epidemiology (STROBE) statement: guidelines for reporting observational studies. Lancet. (2007) 370:1453–7. doi: 10.1016/S0140-6736(07)61602-X, PMID: 18064739

[29] Dupont William D., (1988). Power calculations for matched case-control studies. Biometrics. Available online at: https://www.jstor.org/stable/2531743 (Accessed December 14, 2024) 3233252

[30] Organization WH. Waist circumference and waist-hip ratio: report of a WHO expert consultation, Geneva, 8-11 December 2008. (2011). Available online at: https://apps.who.int/iris/bitstream/handle/10665/44583/?sequence=1 [Accessed December 14, 2024]

[31] Rahman M Berenson AB. Accuracy of current body mass index obesity classification for white, black, and Hispanic reproductive-age women. Obstet Gynecol. (2010) 115:982–8. doi: 10.1097/AOG.0B013E3181DA9423, PMID: 20410772 PMC2886596

[32] Craig CL Marshall AL Sjöström M Bauman AE Booth ML Ainsworth BE . International physical activity questionnaire: 12-country reliability and validity. Med Sci Sports Exerc. (2003) 35:1381–95. doi: 10.1249/01.MSS.0000078924.61453.FB, PMID: 12900694

[33] Cancer AM-NR, (2008) Mechanisms linking physical activity with cancer. Nature Rev Cancer. Available online at: https://www.nature.com/articles/nrc2325 [Accessed December 14, 2024]

[34] Li Y Meeran SM Patel SN Chen H Hardy TM Tollefsbol, TO. Epigenetic reactivation of estrogen receptor-α (ERα) by genistein enhances hormonal therapy sensitivity in ERα-negative breast cancer. Mol Cancer. (2013) 12:1–17. doi: 10.1186/1476-4598-12-9/FIGURES/7 23379261 PMC3577460

[35] Ajabnoor GMA. The molecular and genetic interactions between obesity and breast cancer risk. Medicina (B Aires). (2023) 59:1338. doi: 10.3390/MEDICINA59071338, PMID: 37512149 PMC10384495

[36] Kalantari N Doaei Saeid Keshavarz-Mohammadi Nastaran Gholamalizadeh Maryam Pazan Naeimeh. (2016). Review of studies on the fat mass and obesity-associated (FTO) gene interactions with environmental factors affecting on obesity and its impact on lifestyle. ARYA Atheroscler. Availalbe online at: https://www.ncbi.nlm.nih.gov/pmc/articles/PMC5455327/ [Accessed December 14, 2024] PMC5455327 28607568

[37] Mozafarizadeh M Omran SP Kordestani Z Dehghan HM Faridazar A Houshmand M. Association of obesity-related genetic variants (FTO and MC4R) with breast cancer risk: a population-based case–control study in Iran. Iran J Biotechnol. (2019) 17:e2460. doi: 10.30498/IJB.2019.99594, PMID: 32671127 PMC7357694

[38] Shafiul Hossen M Abdul Barek M Safiqul IM. Obesity and inflammation Lead to insulin resistance and Cancer—a systematic review. Obesity. (2024):39–51. doi: 10.1007/978-3-031-62491-9_3

[39] Danaher J.. Metabolic mechanisms of the fat mass and obesity-associated (FTO) gene. (2016). Available online at: https://vuir.vu.edu.au/34710/ [Accessed December 14, 2024]

[40] Abdollahi S Hasanpour Ardekanizadeh N Poorhosseini SM Gholamalizadeh M Roumi Z Goodarzi MO . Unraveling the complex interactions between the fat mass and obesity-associated (FTO) gene, lifestyle, and Cancer. Adv Nutr. (2022) 13:2406–19. doi: 10.1093/ADVANCES/NMAC101, PMID: 36104156 PMC9776650

[41] Nindrea RD Thongwichian P. FTO gene polymorphisms rs9939609 and the risk of obesity among adults in Western and Asian countries: a systematic review and meta-analysis. Clin Epidemiol Glob Health. (2024) 27:101621. doi: 10.1016/J.CEGH.2024.101621

[42] Younus LA Algenabi AHA Abdul-Zhara MS Hussein MK. FTO gene polymorphisms (rs9939609 and rs17817449) as predictors of type 2 diabetes mellitus in obese Iraqi population. Gene. (2017) 627:79–84. doi: 10.1016/J.GENE.2017.06.005, PMID: 28603074

[43] Doaei S Abdollahi S Mohseni GK Gholamalizadeh M Akbari ME Poorhosseini SM . The effects of FTO gene rs9939609 polymorphism on the association between breast cancer and dietary intake. J Cell Mol Med. (2022) 26:5794–806. doi: 10.1111/JCMM.17595, PMID: 36403211 PMC9716323

[44] Bhardwaj P Au CMC Benito-Martin A Ladumor H Oshchepkova S Moges R . Estrogens and breast cancer: mechanisms involved in obesity-related development, growth and progression. J Steroid Biochem Mol Biol. (2019) 189:161–70. doi: 10.1016/J.JSBMB.2019.03.002, PMID: 30851382 PMC6502693

[45] Park S. L. Cheng Iona Pendergrass Sarah A. Kucharska-Newton Anna M. Lim Unhee Ambite Jose Luis ., (2013). Association of the FTO obesity risk variant rs8050136 with percentage of energy intake from fat in multiple racial/ethnic populations: the PAGE study. Am J Epidemiol. Available online at: https://academic.oup.com/aje/article-abstract/178/5/780/87454 [Accessed December 14, 2024] 10.1093/aje/kwt028 PMC3755639 23820787

[46] Gerard C Brown KA. Obesity and breast cancer–role of estrogens and the molecular underpinnings of aromatase regulation in breast adipose tissue. Mol Cell Endocrinol. (2018) 466:15–30. doi: 10.1016/j.mce.2017.09.014 28919302

[47] Gholamalizadeh M Mirzaei Dahka S Vahid F Bourbour F Badeli M JavadiKooshesh S . Does the rs9939609 FTO gene polymorphism affect fat percentage? A meta-analysis. Arch Physiol Biochem. (2020) 128, 1421–1425. doi: 10.1080/13813455.2020.1773861, PMID: 32574121

[48] Allred D Clark G Elledge R Fuqua SA Brown RW Chamness GC. Association of p53 protein expression with tumor cell proliferation rate and clinical outcome in node-negative breast cancer. J Natl Cancer Inst. Available online at: https://academic.oup.com/jnci/article-abstract/85/3/200/983878 [Accessed December 14, 2024] 10.1093/jnci/85.3.200 8423624

[49] Ehrlich AC Friedenberg FK. Genetic associations of obesity: the fat-mass and obesity-associated (FTO) gene. Clin Transl Gastroenterol. (2016) 7:e140. doi: 10.1038/CTG.2016.1, PMID: 26821195 PMC5543400

[50] Picon-Ruiz M Morata-Tarifa C Valle-Goffin JJ Friedman ER Slingerland JM. Obesity and adverse breast cancer risk and outcome: mechanistic insights and strategies for intervention. CA Cancer J Clin. (2017) 67:378–97. doi: 10.3322/CAAC.21405, PMID: 28763097 PMC5591063

[51] Lake B Damery S Jolly K. Effectiveness of weight loss interventions in breast cancer survivors: a systematic review of reviews. BMJ Open. (2022) 12:e062288. doi: 10.1136/BMJOPEN-2022-062288, PMID: 36207046 PMC9557263

[52] Hess ME Brüning JC. The fat mass and obesity-associated (FTO) gene: obesity and beyond? Biochim Biophys Acta Mol basis Dis. (2014) 1842:2039–47. doi: 10.1016/J.BBADIS.2014.01.017, PMID: 24518103

[53] Romieu I Dossus L Barquera S Hervé MB Paul WF Marc G . Energy balance and obesity: what are the main drivers? Cancer Causes Control. (2017) 28:247–58. doi: 10.1007/s10552-017-0869-z 28210884 PMC5325830

[54] Friedenreich CM Neilson HK Farris MS Courneya KS. Physical activity and cancer outcomes: a precision medicine approach. Clin Cancer Res. (2016) 22:4766–75. doi: 10.1158/1078-0432.CCR-16-0067, PMID: 27407093

